# Studies on antibiotic residues in food of animal origin

**DOI:** 10.1186/2047-2994-4-S1-P168

**Published:** 2015-06-16

**Authors:** A Gaurav

**Affiliations:** 1 Veterinary Public Health, Rajuvas, Udaipur, India

## Introduction

Rapid screening methods (immunological or microbial inhibition assays) are commonly used to detect the presence of antimicrobials in food but more accurate chromatographic methods are required to identify and confirm the presence of these compounds.

## Objectives

Keeping in view the consumer safety and public health significance of antibiotic residues in milk, the present study was conducted to screen milk samples for the presence of antibiotic residues.

## Methods

The residual levels of tetracyclines, fluoroquinolones, aminoglycosides, chloramphenicol and sulfamethazine in raw milk samples from Punjab were monitored by ELISA.

## Results

It was found that out of 133 milk samples analyzed, 18 samples were found to contain tetracycline residues. The concentrations of tetracycline residues in cattle milk samples were in the range 16-134.5 ppb. Similarly, out of 60 cattle milk samples analyzed, 6 samples were found positive for fluoroquinolone (enrofloxacin/ciprofloxacin) residues, while 4 samples were found positive for aminoglycoside (streptomycin/dihydrostreptomycin) residues giving a prevalence rate of 10% and 6.67%, respectively. Out of 107 milk samples analysed from 4 districts of Punjab viz., Amritsar, Ferozpur, Muktsar and Ludhiana, chloramphenicol was not detected in any of the milk samples. While out of 72 milk samples analysed from 3 districts of Punjab viz., Muktsar, Ferozpur and Ludhiana, sulfamethazine was detected in three samples. The maximum and minimum residue levels for sulfamethazine detected were 292.94 μg/kg and 29.19 μg/kg, respectively.

## Conclusion

ELISA based detection of antibiotic residues revealed 13.5, 10, 6.67, 0 and 4.16% positive samples for tetracyclines, fluoroquinolones, aminoglycosides, chloramphenicol and sulfamethazine, respectively.

## Disclosure of interest

None declared.

